# Safety and accuracy of AI in triaging patients in the emergency department

**DOI:** 10.1186/s12245-025-01069-x

**Published:** 2025-11-20

**Authors:** Lama Mohammad Alomari, Mai Mamdouh Alshammari, Asal Osama Arbaeen, Raghad Abdullah Alshehri, Hanin Saad Almalki

**Affiliations:** 1 Emergency Department, King Saud Medical City, Riyadh, Saudi Arabia; 2 Emergency Medicine, King Saud Medical City, Riyadh, Saudi Arabia; 3 Emergency Medicine, Prince Mohammed bin Abdulaziz Hospital, Riyadh Second Health Cluster, Riyadh, Saudi Arabia

**Keywords:** Emergency department, AI, Triage, ChatGPT

## Abstract

**Background:**

Artificial Intelligence (AI) has been increasingly explored in healthcare, particularly in emergency department (ED) triage. This study aimed to evaluate the effectiveness of the AI chatbot ChatGPT in triaging patients, focusing on its accuracy, safety, efficiency, and impact on patient care.

**Methods:**

A prospective observational study was conducted at the ED of King Saud Medical City (KSMC) in Riyadh, Saudi Arabia, with a sample size of 138 patients. Patients requiring immediate resuscitation were excluded. ED physicians assigned triage scores using the Canadian Triage and Acuity Scale (CTAS), followed by AI-generated scores for the same patients. In cases of discrepancy, the final decision by the senior ED consultant was considered the gold standard. The study assessed inter-rater reliability between AI and human raters and evaluated the accuracy of each compared to the consultant’s assessment.

**Results:**

The results indicated a high agreement rate (85.61%) between ChatGPT and ED physicians, with substantial inter-rater reliability (κ = 0.780, 95% Confidence Interval [CI] 0.676–0.884, *p* < 0.001). Agreement between ED physicians and consultants was at 63.9%, with moderate reliability (κ = 0.406, 95% CI 0.006–0.806, *p* = 0.018). Consultants assigned lower acuity levels than physicians in most cases. ChatGPT’s accuracy compared to the consultant was 42.86%, with slight reliability, showing a tendency to overestimate acuity, particularly in critical cases. However, it performed better in mid-range acuity levels.

**Conclusion:**

The findings suggested that AI could support ED triage by aligning closely with human decision-making. However, its overestimation of severity could lead to over-triaging and increased resource use. Limitations included a small sample size and the use of a general AI model not specifically trained for medical triage. Future research should focus on AI models tailored for ED triage to improve reliability and clinical applicability.

## Introduction

Triage is a crucial process in emergency medicine. It involves prioritizing patients based on the severity of their condition. The primary goal of triage is to ensure that the most critically ill patients receive prompt attention to maximize their chances of survival and reduce overall morbidity and mortality rates. There are several scales for triaging patients in emergency medicine. Some of the most used scales include the Emergency Severity Index (ESI), the Canadian Triage and Acuity Scale (CTAS), the Manchester Triage System (MTS), and Simple Triage and Rapid Treatment (START).

All triage scales help healthcare providers prioritize patients and ensure efficient allocation of resources in emergency situations. However, healthcare providers may encounter two significant challenges highlighted as over-triage and under-triage. Over- triage occurs when patients with less severe conditions are categorized as high priority, leading to unnecessary interventions, and diverting attention away from patients with more urgent conditions. On the other hand, under-triage occurs when patients with critical conditions are assigned a lower priority, delaying the necessary treatment, and increasing the risk of morbidity and mortality. Proper triaging is essential to optimize patients’ outcomes, resource allocation, and the overall functioning of the emergency medicine system. Several strategies are used to mitigate the risk of under-triage and over-triage and to ensure proper triaging. Strategies include using clear triage protocols, regular training, and continuous monitoring.

Hospitals in Saudi Arabia are working in a collaborative ecosystem termed as Health Clusters. Our hospital is the receiving hospital for emergency cases in Riyadh’s First Health Cluster. This gives our hospital the authority to direct less urgent cases to emergency departments for other hospitals working under the umbrella of the cluster, which helps in the efficient allocation of resources and reduces waiting times in our ED. Artificial Intelligence (AI) implementation in the healthcare sector has been evolving over the last few years. Machine learning technologies can analyze a large amount of healthcare data, identify patterns, and predict outcomes, which would play a key role in preventing Emergency Department (ED) overcrowding and overcoming staff shortages if used as a tool for triaging patients.

Studies in the literature have consistently demonstrated the potential benefits of using AI in different healthcare settings, including triaging patients in the ED. A Turkish study assessed the performance of an AI chatbot application (ChatGPT) in triaging patients and showed poor performance except for level 1 and level 2 cases [1]. This result of poor performance was parallel to what was reported in French and Korean studies [2, 3]. The Korean study showed better accuracy with higher-level cases in contrast to the previously mentioned study, along with differences in the performance of different ChatGPT versions [3]. On the other hand, another Turkish study showed perfect agreement between ChatGPT triaging decisions and ED triage team decisions [4].

Implementation of AI in triaging ED patients is relatively a new area of interest with inconsistent results in the literature. Researchers are interested in conducting studies to assess its triaging accuracy, as its usage would help in reducing wait times and ED overcrowding and optimizing human resources allocation, ultimately improving patients’ outcomes. Given the fact that our ED is one of the busiest EDs in Saudi Arabia, we aimed to evaluate the effectiveness of the AI chatbot application (ChatGPT) in triaging patients, with a focus on its accuracy, safety, efficiency, and overall impact on patient care.

## Methods

This was a prospective observational study conducted at the Emergency Department (ED) at King Saud Medical City (KSMC), Riyadh, Saudi Arabia. Our aim was to evaluate the triaging decision of Artificial Intelligence (AI) and compare it to the emergency physician’s decision. This evaluation was performed in two steps. Initially, patients presenting to our ED were triaged by the emergency physician assigned in triage as usual. Following that, a research assistant documented the necessary information from the same patient on a standard data collection sheet. This data was then entered into a specified AI program, and its triaging decision was recorded. Next, if the AI’s triaging decision differed from that of the emergency physician, the research assistant referred the case to the Emergency Medicine consultant on duty (the gold standard) to review the patient’s information and provide their assessment. This step was carried out without disclosing the AI and ED physician’s triaging decisions to the consultant. Based on the collected data, we measured the inter-rater reliability between the emergency physician and AI using Cohen’s Kappa coefficient to determine the level of agreement between the two raters. Cases with triage outcome discrepancies underwent inter-rater reliability measurement again, with each rater being compared to the gold standard to determine their level of accuracy.

In this study, we used ChatGPT based on OpenAI’s GPT- 4.0 model, a frequently used and supervised machine learning-based chatbot. The data collection sheet contained the patient’s demographics, vital signs at presentation, chief complaint, and the triaging decisions of both the physicians and ChatGPT. All patients were presented to ChatGPT in the form of short, de-identified, unified clinical descriptions (age, sex, comorbidity, presenting complaint, vital signs, and relevant context as for example GCS or AVPU) followed by the clear instruction: “Based on CTAS, what category does this patient fall into?”. In our local clinical context, the term ED physician in this study specifically refers to frontline registrars (senior residents) providing initial triage in the emergency department. The emergency consultants involved in this study as the gold standard were board-certified senior physicians with at least five years of experience accredited by Saudi Commission for Health Specialties (SCFHS). The official triaging score used was the Canadian Triage and Acuity Scale (CTAS). All patients presenting to our ED were considered eligible, except for those in cardiac arrest who required immediate resuscitation or intervention, as they were excluded. Eligible patients were enrolled following their consent to participate, which was obtained by the research assistant and documented in each data collection sheet. Data collection continued until we reached 138 patients, based on a predetermined sample size calculation powered to achieve statistical significance with a 5% margin of error.

### Statistics

Statistical analysis was performed using the Statistical Package for Social Sciences (SPSS). The primary objective was to measure the inter-rater reliability between human and AI triage decisions. The secondary objective was to determine the accuracy of each rater by comparing their assessment to the gold standard in cases of discrepancy. Inter-rater reliability was measured using Cohen’s Kappa coefficient, where values ≤ 0 indicated no agreement, 0.01–0.20 indicated slight agreement, 0.21–0.40 indicated fair agreement, 0.41–0.60 indicated moderate agreement, 0.61–0.80 indicated substantial agreement, and 0.81–1.00 indicated almost perfect agreement.

## Results

Table 1 summarizes the demographic characteristics and medical history of the patients studied. Most patients were male (61.2%). The largest age group was 36–45 years (20.9%), followed closely by the 26–35 age group (19.4%), with the fewest patients younger than 18 years (5.0%).

**Table 1:** Demographic and clinical characteristics of study patients

Variable	Category	*N*	%
Mode of arrival	EMS	18	12.9
	Personal vehicle	121	87.1
Companion present	No	13	9.4
	Yes	126	90.6
Patient ambulatory	No	28	20.1
	Yes	111	79.9
Age	< 18	7	5.0
	18–25	20	14.4
	26–35	27	19.4
	36–45	29	20.9
	46–55	19	13.7
	56–65	17	12.2
	> 66	20	14.4
Gender	Female	54	38.8
	Male	85	61.2
Comorbidities	Diabetes mellites	31	22.3
	Hypertension	27	19.4
	Asthma	6	4.3
	Benign Prostatic Hyperplasia	4	2.9
	Ischemic Heart Disease (IHD)	8	5.8
	End-Stage Renal Disease (ESRD)	3	2.2
	Cancer	3	2.2
	Chronic Kidney Disease (CKD)	3	2.2
	Heart Failure (HF)	4	2.9
	None	65	46.8
	Unknown	11	7.9
Any known allergies	No	117	84.2
	Yes	3	2.2
	unknown	19	13.7

This table summarizes demographics, mode of arrival, ambulation status, and common comorbidities among the 138 patients enrolled

N: number of patients; percentages represent proportions within each category

The clinical presentation and chief complaints of patients in the study are shown in Table 2. Most cases were classified as non-trauma presentations (77.0%).

**Table 2:** Clinical presentation and chief complaints

Variable	Category	*N*	%
Type of clinical presentation	Non-Trauma	107	77.0
	Trauma	32	23.0
Chief complaints	Abdominal pain	7	5.0
	Burns	4	2.9
	Chest pain	10	7.2
	Confusion / Altered consciousness	5	3.6
	Cough	6	4.3
	Dizziness / Vertigo	7	5.0
	Dysuria	3	2.2
	Eye problems	5	3.6
	Facial trauma	4	2.9
	Fever	3	2.2
	Finger/hand injury	6	4.3
	Foot injury	4	2.9
	Foreign body / puncture wound	2	1.4
	Generalized body pain	2	1.4
	Head trauma	7	5.0
	Headache	4	2.9
	Hematuria	2	1.4
	Infection (skin, surgical wounds, bedsores)	6	4.3
	Jaundice / abdominal pain (suspected cholangitis)	4	2.9
	Leg / Lower limb swelling	3	2.2
	Lower back pain	4	2.9
	Musculoskeletal pain (joint, shoulder, ankle)	5	3.6
	Nausea / Vomiting	3	2.2
	Palpitations	4	2.9
	Rash / Allergy	2	1.4
	Respiratory distress / SOB	9	6.5
	Seizure	3	2.2
	Trauma (unspecified/falls/RTAs)	11	7.9
	Urinary retention	3	2.2
	Weakness / numbness	4	2.9
	Others	6	4.3

This table presents the frequency and types of trauma versus non-trauma cases, along with patient-reported chief complaints at ED triage. Each patient may report more than one symptom

N: Number of patients; percentages represent proportions within each category

Table 3 summarizes the patients’ general appearance and pain assessment at the time of presentation. Most patients had a normal appearance (59.0%), while approximately a quarter appeared to be in pain (24.5%).

**Table 3:** Patient general appearance and pain severity assessment

Variable	Category	*N*	%
Patients’ General Appearance	None (Normal appearance)	82	59.0%
	In pain	34	24.5%
	Drowsy	11	7.9%
	In respiratory distress	10	7.2%
	Diaphoretic	1	0.7%
	Jaundiced	1	0.7%
Pain Score	Mild pain (1–3)	91	65.5%
	Moderate pain (4–6)	37	26.6%
	Severe pain (7–10)	11	7.9%

This table shows clinical impressions (e.g., pain, drowsiness) and self-reported pain scores using the standard numeric scale (0–10). Percentages reflect proportion of total patient sample

N: Number of patients; percentages represent proportions within each category

Table 4 presents the vital signs and clinical measurements of the patients upon presentation. Most patients had normal heart rates (73.4%), while approximately a quarter presented with tachycardia (25.2%) and only a few with bradycardia (1.4%). Blood pressure readings were normal in (36.0%) and hypertensive in (59.7%), and hypotensive in a small proportion (4.3%).

The majority of ECG interpretations (77.0%) were unknown or undocumented. Among documented ECG findings, normal sinus rhythm was most common (12.2%), while non-specific ST/T-wave changes (2.9%) and atrial fibrillation (2.2%) were the next most frequent abnormalities.

Similarly, VBG interpretations were largely undocumented or unavailable (76.2%). When available, the most frequent interpretation was normal (11.5%), followed by metabolic acidosis (4.3%), mixed acid-base disorders (3.6%), electrolyte abnormalities (2.2%), and extremely abnormal (critical) values (0.7%).

**Table 4:** Vital signs and clinical measurements

Variable	Category	*N*	%
Heart rate	Bradycardia	2	1.4
	Normal	102	73.4
	Tachycardia	35	25.2
Blood pressure	Hypotension (Low BP)	6	4.3
	Normal	50	36.0
	Hypertension	83	59.7
Respiratory rate	Normal	108	77.7
	Tachypnea	31	22.3
Oxygen Saturation (SpO₂) Classification on Room Air	Normal	126	90.6
	Hypoxia	13	9.3
Body Temperature	Normal	129	92.8
	fever	10	7.2
Random Blood Sugar	Hypoglycemia	1	0.7
	Normal	128	92.1
	Hyperglycemia	10	7.2
AVPU Category based on GCS Score	Alert (A)	130	93.6
	Verbal Response (V)	7	5.0
	Pain Response (P)	1	0.7
	Unresponsive (U)	1	0.7
ECG Interpretation	Unknown / Not Documented / Unavailable	107	77.0
	Normal Sinus Rhythm (Normal ECG)	17	12.2
	Atrial Fibrillation (AFib / AFib RVR)	3	2.2
	Left Bundle Branch Block (LBBB)	1	0.7
	Right Bundle Branch Block (RBBB)	1	0.7
	Left Ventricular Hypertrophy (LVH)	3	2.2
	Sinus Bradycardia	1	0.7
	Supraventricular Tachycardia (SVT)	1	0.7
	ST/T-wave changes (Non-specific)	4	2.9
	Q-wave abnormalities	2	1.4
Venous Blood Gas (VBG) Interpretation	Unknown / Not Documented / Unavailable	106	76.2
	Normal VBG	16	11.5
	Metabolic Acidosis (including lactate)	6	4.3
	Electrolyte Abnormalities	3	2.2
	Mixed Acid-base Disorders	5	3.6
	Extremely Abnormal (Critical Values)	1	0.7
	Unremarkable (unspecified)	2	1.4

This tables includes vital signs and AVPU score. Also summarizes documented ECG and VBG findings. “Normal” values are based on standard adult reference ranges

N: Number of patients; percentages represent proportions within each category

The agreement between ED Physician and ChatGPT triage scores using the CTAS are shown in Table 5. The overall accuracy of agreement was high at 85.61%, with a substantial inter-rater reliability indicated by a Kappa (κ) value of 0.780 (95% CI 0.676–0.884, *p* < 0.001). The ED physician’s triage ratings closely aligned with ChatGPT’s assessments across all CTAS categories, especially in categories 2, 3, and 4, where agreement ranged from 83.9% to 86.8%. Category 5 showed perfect agreement, although with very few cases. This suggests that ChatGPT’s performance in assigning CTAS scores closely mirrors the ED physician’s triage decisions.

**Table 5:** Agreement between ED physicians and ChatGPT triage scores using CTAS

ED Physicians CTAS Score	ChatGPT CTAS Triage Score						Kappa (κ) [95% CI]	Overall accuracy	p-value
	1	2	3	4	5	Total			
**2**	4 (6.5%)	52 (83.9%)	6 (9.7%)	0 (0%)	0 (0%)	62 (100%)	0.780 [0.676–0.884]	85.61%	< 0.001
**3**	0 (0%)	2 (3.8%)	46 (86.8%)	5 (9.4%)	0 (0%)	53 (100%)			
**4**	0 (0%)	0 (0%)	2 (9.5%)	18 (85.7%)	1 (4.8%)	21 (100%)			
**5**	0 (0%)	0 (0%)	0 (0%)	0 (0%)	3 (100%)	3 (100%)			
**Total**	4 (2.9%)	54 (38.8%)	54 (38.8%)	23 (16.5%)	4 (2.9%)	139 (100%)			

This table displays agreement between ED physicians and ChatGPT across CTAS categories. Each row corresponds to physician-assigned CTAS, with percentages representing distribution of ChatGPT’s triage scores within each physician-assigned group. Inter-rater reliability measured via Cohen’s kappa and overall accuracy

The agreement between the ED Physician and the ED Consultant (Gold standard) in assigning CTAS triage scores is shown in Table 6. The overall accuracy was 63.9%, with a moderate inter-rater reliability indicated by a Kappa (κ) value of 0.406 (95% CI 0.006–0.806, *p* = 0.018). There were noticeable discrepancies between the two raters, particularly in the categorization of acuity levels. For patients assigned CTAS 2 by the ED Physician, only 50% matched the Consultant’s classification, while the remaining 50% were classified as CTAS 3. Among cases rated as CTAS 3 by the Physician, 75% aligned with the Consultant, but 25% were considered CTAS 4. Similarly, one-third of cases assigned CTAS 4 by the Physician were rated as CTAS 3 by the Consultant. The ED Consultant most frequently categorized patients as CTAS 3 (57.1%), followed by CTAS 2 (23.8%) and CTAS 4 (19.4%).

**Table 6:** Agreement between ED physicians and ED consultants (Gold standard) on CTAS scores (*n* = 21)

ED Physicians CTAS Score	ED Consultants CTAS Score (Gold standard)				Kappa (κ) [95% CI]	Overall accuracy	p-value
	CTAS 2	CTAS 3	CTAS 4	Total			
2	5 (50%)	5 (50%)	0 (0%)	10 (100%)	0.406 [0.006–0.806]	63.9%	0.018
3	0 (0%)	6 (75%)	2 (25%)	8 (100%)			
4	0 (0%)	1 (33.3%)	2 (66.7%)	3 (100%)			
Total	5 (23.8%)	12 (57.1%)	4 (19.4%)	21 (100%)			

This table presents cross-tabulated CTAS scores assigned by ED physicians and consultants for a subset of 21 cases. Inter-rater reliability metrics (kappa, 95% confidence interval, overall accuracy, and p-value) are reported

The agreement between the ED Consultant (Gold standard) and ChatGPT in assigning CTAS triage scores is shown in Table 7. The overall accuracy was 42.86%, with a slight level of inter-rater reliability indicated by a Kappa (κ) value of 0.168 (95% CI -0.26-0.599, *p* = 0.004). While ChatGPT demonstrated moderate agreement in some categories, discrepancies were notable. All cases classified as CTAS 1 by ChatGPT were assessed as CTAS 2 by the Consultant, reflecting consistent overestimation. In contrast, ChatGPT correctly matched 7 out of 9 cases in the CTAS 3 category and 2 out of 5 in CTAS 4. The single CTAS 5 case by ChatGPT was accurately matched.

**Table 7:** Agreement between ChatGPT and ED consultants (Gold standard) on CTAS scores (*n* = 21)

ChatGPT CTAS Triage Score	ED Consultants CTAS Score (Gold standard)				Kappa (κ) [95%CI]	Overall accuracy	p-value
	CTAS 2	CTAS 3	CTAS 4	Total			
1	4 (100%)	0 (0%)	0 (0%)	4 (100%)	0.168 [-0.26-0.599]	42.86%	0.004
2	0 (0%)	2 (100%)	0 (0%)	2 (100%)			
3	1 (11.1%)	7 (77.8%)	1 (11.1%)	9 (100%)			
4	0 (0%)	3 (60.0%)	2 (40%)	5 (100%)			
5	0 (0%)	0 (0%)	1 (100%)	1 (100%)			
Total	5 (23.8%)	12 (57.1%)	4 (19%)	21 (100%)			

This table compares ChatGPT-assigned CTAS scores to those of ED consultants across 21 discrepant cases. Highlights tendency of over-triage by ChatGPT in high-acuity categories. Statistical agreement measures are presented

## **Discussion**

AI is transforming emergency services by enhancing various aspects of patient care, from diagnosis and imaging analysis to decision-making and resource allocation [5]. Its applications extend to predicting clinical outcomes, integrating patient monitoring systems, addressing challenges such as ED overcrowding and optimizing triage processes [6, 7]. AI-driven triage systems can facilitate rapid and effective patient categorization based on severity, complementing the essential role of triage nurses in ensuring timely treatment [7, 8]. The primary objective of this study was to measure inter-rater reliability between human ED physicians and an AI system (ChatGPT) using the CTAS. In this study, the triage performance of ChatGPT compared to ED physicians and consultants reveals important patterns in agreement, direction of triage decisions, and implications for clinical safety. Table 5 shows a high level of agreement between ChatGPT and ED physicians, with an overall accuracy of 85.61% and a substantial inter-rater reliability (κ = 0.780, 95% CI 0.676–0.884, *p* < 0.001). Discrepancies between ChatGPT and ED physicians were generally limited to adjacent CTAS levels, occurring in both directions. For instance, ChatGPT over-triaged 6.5% of CTAS 2 patients to CTAS 1, while under-triaging 9.7% to CTAS 3. This balanced pattern suggests that ChatGPT neither consistently overestimated nor underestimated acuity compared to ED physicians, but rather demonstrated a pattern of cautious variability within acceptable clinical range with high overall agreement that aligns with prior research suggesting AI’s potential to replicate clinician assessments closely [9, 10]. While the high level of agreement between ED physicians and ChatGPT appears promising, it is also crucial to interpret this in light of the discrepancies revealed when comparing both against the gold standard of the ED consultant as different trend emerges. Table 6 compares ED physicians with ED consultants. The overall accuracy dropped to 63.9%, and the inter-rater reliability was moderate (κ = 0.406, 95% CI 0.006–0.806, *p* = 0.018). Importantly, physicians more frequently assigned higher acuity levels than the consultant, particularly in CTAS 2 and 3 cases. For example, 50% of patients initially assigned CTAS 2 by physicians were downgraded to CTAS 3 by the consultant, and 25% of CTAS 3 cases were downgraded to CTAS 4. This trend suggests a tendency toward over-triage by ED physicians compared to the gold standard, likely reflecting risk-aversion clinical behavior, potentially prioritizing patient safety over resource optimization with consistent results in the literature [11, 12].

Table 7 highlights ChatGPT’s performance relative to the consultant, where the overall accuracy declined further to 42.86%, with slight inter-rater reliability (κ = 0.168, 95% CI -0.26-0.599, *p* = 0.004). Unlike its pattern with physicians, ChatGPT tended to assign higher acuity levels than the consultant, particularly evident in CTAS 1 cases. All four patients initially assigned CTAS 1 by ChatGPT were reassessed as CTAS 2 by the consultant. This reflects ChatGPT’s tendency to overestimate severity compared to the gold standard, particularly in high-acuity scenarios. This overestimation has been reported in prior literature, especially among senior physicians [13–16]. This low alignment with expert-level assessment reflects falter performance in the nuanced interpretation of clinical severity, particularly when not directly trained on gold-standard validated datasets.

These findings carry important implications for safety. While over-triage can increase resource use and ED burden, it is generally safer than under-triage, which risks delayed care. ChatGPT’s pattern of overestimating severity in comparison to the consultant may thus be interpreted as a safety-preserving bias, albeit one that could strain ED workflow if not calibrated. The discrepancy in ChatGPT’s alignment—strong agreement with ED physicians but only slight agreement with consultants—likely reflects shared interpretation styles or biases between human clinicians and the AI rather than correct classification. Masanneck et al. found similar result of strong alignment between ChatGPT and professionally trained ED staff (κ = 0.67) in comparison to lower alignment with expert-level consultants [14]. This phenomenon is consistent with known biases documented in machine learning models trained on clinical decisions, which replicate rather than resolve human inaccuracies [17]. Overall, while ChatGPT demonstrated high concordance with ED physicians, its lower agreement with the gold standard and its tendency to overestimate acuity suggest that it should currently be viewed as a supportive tool, not a standalone triage decision-maker which is supported by the literature [18–20]. Ongoing refinement and validation using consultant-rated data are necessary to improve its clinical reliability and safety in emergency triage.

This study has had several strengths, most notably the powered sample size, the use of the structured data collection sheet, and the concealment of the decision of triage when presented to the gold standard to prevent bias. However, there were a few notable limitations. Although our sample size was powered to detect statistical significance, it was still considered a small sample size which may pose a challenge when replicating the findings, potentially impacting generalizability. Of note, the use of a general AI model not specifically trained for medical triage could be a contributing factor. Additionally, this was an observational study which may be subject to confounding factors that were not fully controlled as ED triage physician’s clinical experience, case complexity and missing clinical data (e.g., ECG/VBG) might have influenced the ChatGPT’s decision in some cases.

## Conclusion

In this study, we found a significantly high level of agreement between ED physicians and ChatGPT, indicating substantial inter-rater reliability. Integrating AI into emergency medicine holds significant promise, but realizing this potential requires ongoing research, rigorous evaluation, and a thoughtful approach to implementation. It is essential to prioritize patient safety and ensure that AI systems are used responsibly and effectively to enhance, not replace, the expertise of human clinicians. Future research should focus on AI models tailored for ED triage to improve reliability and clinical applicability.

## Data Availability

The datasets used and/or analyzed during the current study are available from the corresponding author on reasonable request.
